# Channel Occupancy Measurements in 868 MHz ISM Band in Residential Areas

**DOI:** 10.3390/s21237805

**Published:** 2021-11-24

**Authors:** Sebastian Kozłowski, Krzysztof Kurek

**Affiliations:** Institute of Radioelectronics and Multimedia Technology, Warsaw University of Technology, ul. Nowowiejska 15/19, 00-665 Warsaw, Poland; S.Kozlowski@ire.pw.edu.pl

**Keywords:** IoT, channel occupancy, ISM band

## Abstract

The rapid development of Internet of Things (IoT) has led to more and more devices using ISM frequency bands. Because they are not time synchronized, medium access collisions are unavoidable. The probability of such a collision is usually reasonably low; however, it increases with the number of transmitters competing for the same frequency channel. For this reason, ISM bands’ occupancy is regularly monitored by researchers. This paper presents the results of the measurement campaign during which a selected part of the 868 MHz ISM frequency band was monitored for the presence of transmissions in six locations in various residential areas in Warsaw, Poland. For the purpose of the campaign, a dedicated measurement set-up comprising a software-defined radio (SDR) module was assembled. The measurements results showed that the channel occupancy is in most cases lower than 1% with a maximum observed value of 2%. The paper presents selected characteristics of the detected signals. Additionally, distribution over time of the detected signals was used together with the Monte Carlo simulations to analyze how long idle time blocks are available for new transmitters that could be deployed in the band under testing.

## 1. Introduction

Nowadays, a rapid development of Internet of Things (IoT) with the use of wireless communication is observed. Both the number of operating IoT networks and the number of IoT devices within each single network are growing and in the future systems with millions of devices/sensors per km^2^ are foreseen [1].

IoT solutions may operate either in licensed (LTE-M, NB-IoT) or ISM (LoRa, Sigfox, Wireless M-Bus) frequency bands. The latter is often chosen as the better option [2,3,4,5,6] for many reasons, including cost, flexibility, and independence from third-party operators. However, unlicensed bands are available for everybody under some limitations regarding transmitter characteristics such as power and duty cycle [7]. Consequently, in a typical situation many devices operate in an ISM band without any time synchronization, which leads to intra- and intersystem interferences. Medium access is realized basing on the assumption that if transmitted data packets are short and the intervals between them are long enough, the medium access collision probability is reasonably low.

Such an approach works well as long as the number of devices using the same frequency does not exceed a certain limit. This implies that deploying a new or expanding an existing wireless system operating in an ISM band may disturb other systems already operating in the same area. In order to predict if such problems may occur in the near future, it is necessary to monitor how intensively unlicensed bands are utilized and, given the aforementioned rapid development of IoT, such monitoring should be repeated at reasonable intervals.

Spectrum occupancy measurements present numerous challenges which were addressed by many researchers. In a wideband approach [8,9,10,11,12,13,14], a set of dedicated antennas, and sometimes also a set of dedicated RF front-ends, is required to cover different frequency sub-bands. In order to obtain reliable statistics, each frequency must be observed for a sufficiently long time period. If a part of the set-up is common for all sub-bands (e.g., a spectrum analyzer) and in consequence the measurements for the sub-bands cannot be carried out simultaneously, the entire campaign takes a long time. Additionally, it is also necessary to identify and exclude from the analysis frequency ranges in which spread-spectrum transmissions are expected. Otherwise, misleading conclusions could be drawn. Good illustrations of this problem were indicated in [9], where 3G cellular network signals remained undetected, and in [10], where the measurements showed practically no occupation of the band around 1575 MHz, while this particular band is actually continuously occupied by Global Positioning System (GPS) L_1_ signal.

In other approaches, the frequency range used or intended to be used in the selected application is subjected to measurements and analysis [15,16,17,18,19,20,21,22,23]. In this case, researchers give particular attention to 2.4 GHz and 5 GHz ISM bands, widely used by WLAN devices [17,18,19,20,21,22,23]. 

In this paper, we focus on the 868 MHz ISM band used in Europe by IoT systems such as LoRa, Sigfox, or Wireless M-Bus. The literature survey allows one to conclude that the use of this part of the radio spectrum was studied less intensively than it was for the two bands mentioned above. 

In [24], the utilization of the 865–868 MHz sub-band by RFID tag readers was measured to determine their impact on potential IoT devices without referring to any specific IoT system. The obtained occupancy values range from 0 to 80%, depending on the measurement location. 

In [25], the power levels of detectable signals were measured in five different locations (residential, industry, hospital, business, and shopping areas) in Aalborg, Denmark, in 2017. The results showed that the average occupancy in the 868–869 MHz range was about 20–30% for business and shopping areas, but below 3% for industrial and residential areas. In [26], the results from [25] were used to determine the influence of external interferences on the performance of LoRa and Sigfox systems in outdoor and indoor scenarios. The conclusion of the paper was that the interferences can reduce coverage up to 10% for outdoor scenarios and up to 20–50% for indoor ones, and, furthermore, transmission failure rate may increase to 50% for LoRa and about 60% for Sigfox. 

In [27], short and long term measurements of the spectrum occupancy in 868 MHz band carried out in two urban locations in 2013 were presented. The results showed that the occupancy strongly depends on the frequency within the monitored frequency range and for certain frequencies may reach 80%, whereas other parts of the spectrum under test might not be used at all. Obtained measurement results were also used to develop a statistical model of transmission distribution over time for a selected fragment of the monitored spectrum. The model was then used for determining the medium access collision probability.

The short review of the above works shows that the occupancy of the 868 MHz band can vary within a fairly wide range, depending on the location, the type of environment, and particular sub-band.

The contribution of the present paper can be summarized as follows. First, we have provided new results of spectrum occupancy measurements obtained in 2021 in a few apartments located in residential areas. Secondly, we have also presented the structure of the recorded transmissions, including their duration, repeatability, and representative time-domain waveforms. On the basis of several examples, we have indicated that the abovementioned data, which could be collected owing to the adopted measurement technique and the use of a dedicated system, may provide some information about the origin of individual transmissions, but this issue requires more advanced analysis. Finally, we have used the knowledge about the distribution of the recorded transmissions over time to analyze the gaps between consecutive transmissions and to determine idle time available for potential new devices. In contrast to [27], we used Monte Carlo simulations supported by the measurement data to investigate two cases: when only intentional transmissions are taken into consideration as the interferences and when all recorded transmissions are taken into consideration as the interferences.

## 2. Measurement Methodology

### 2.1. Measurement Set-Up

**Figure 1:**
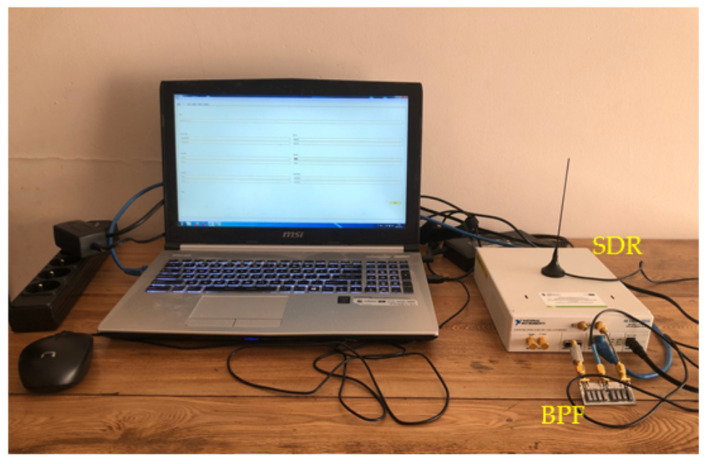
The photograph of the measurement set-up.

The measurement set-up, shown in Figure 1, consists of an SDR module NI USRP 2931 [28] communicating with the laptop computer via Gigabit Ethernet (GbE) interface. Radio signals are received using a simple monopole antenna and provided to the SDR module via the bandpass filter (BPF) to attenuate, at least partially, signals from outside the band of interest which may saturate the input amplifier and thus affect the measurements.

It should be noticed that the antenna used was always oriented vertically in relation to the ground surface. Since polarizations of transmitters operating in the area of interest were not guaranteed to be vertical and even vertically emitted waves may have been depolarized due to reflections [29], it must be assumed that only a part of the overall incoming signals’ energy was collected. Additionally, the antenna was not calibrated, i.e., its gain was not known. This is why absolute power levels provided in the paper correspond to the input port of the filter.

The second of the aforementioned factors (unknown gain) does not disturb the relation between the levels of signals originating from different transmitters in the sense that the antenna affects all the signals equally, although it is likely that in a set-up provided with an antenna of similar radiation pattern, but lower losses, more signals would exceed the detection threshold (see Section 2.2 for more details). As far as the polarization is concerned, a linearly polarized monopole obviously may affect different incoming signals in a different way and this effect was not considered in the presented analysis.

### 2.2. Raw Data Processing

The main task of any SDR module is to provide a sample stream corresponding to the baseband representation of the received radio signal to enable user-defined processing necessary for retrieving information carried by the signal. In the case of NI USRP modules, it is easy to store received samples in a file for further processing, either using a self-made application or commonly available tools such as GNU Radio [30]. The problem is usually the amount of data to be stored. For a sampling rate of only 1 MSamples/second, a one-hour-long observation generates almost 30 GB of data, assuming 2 single-precision numbers for representing real and imaginary parts of a sample. Because, according to the previous studies, channel usage is expected to be far less than 100%, most of the raw data corresponds to noise. Taking the above into consideration, for the purpose of the research being the subject of this paper, a dedicated application was implemented in C++. It performed two main functions: storing in a text file basic information regarding the not-noise signal (referred to as “a burst”) and recording in a binary file samples of the first few bursts for eventual visual inspection by the user.

A burst was defined as a fragment of the signal starting from a sample exceeding a predefined threshold and ending with a sample after which for a predefined time no sample exceeds the threshold. A sample is considered as exceeding the threshold if either its real or imaginary part exceeds the threshold. According to this definition, a short gap between two consecutive sets of high-amplitude samples is ignored. The introduction of such a principle was necessary to prevent the algorithm from detecting particular symbols as separate bursts in the case of amplitude modulation schemes, such as on-off keying. Consequently, in the resulting text file, a single detected burst is described with a log entry comprising four numbers: start time, length, mean power, and duty factor; mean power being defined as the sum the squared amplitudes of the samples divided by the total number of samples in the burst, and the duty factor being defined as the ratio of the number of samples exceeding the threshold and the total number of samples in the burst. The duty factor of the burst should not be confused with the duty cycle of the transmitter. Using such a format, one needs usually less than 2 MB to log the data collected during the one-hour observation period.

### 2.3. Measurement Set-Up Calibration

The threshold value was assumed to be twice the maximum noise amplitude observed during a few-seconds-long time period. The gain of the receiving chain of the SDR module was always set to its maximal level of 38 dB. To determine the relationship between the unitless amplitude obtained from the SDR module and real signal level expressed in dBm, a sine wave of known adjustable power was provided to the input of the bandpass filter BPF to which normally the antenna is connected. It was determined that correction coefficient equals 40 dB, i.e., the actual signal power expressed in dBm equals the power calculated from the raw data minus 40 dB. 

### 2.4. Measurement Locations and Frequency Bands of Interest

**Figure 2:**
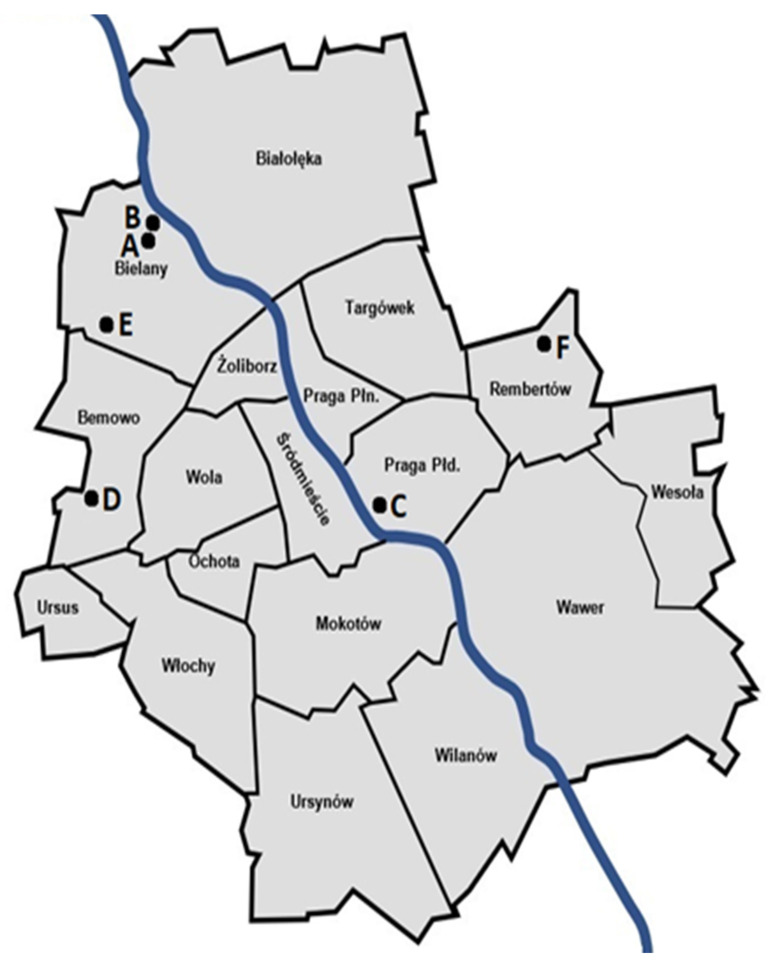
Measurement locations on the map of Warsaw.

The measurements were carried out in six locations, denoted as A, B, C, D, E, and F. All the locations are private apartments in various residential districts of Warsaw, Poland (Figure 2).

**Figure 3:**
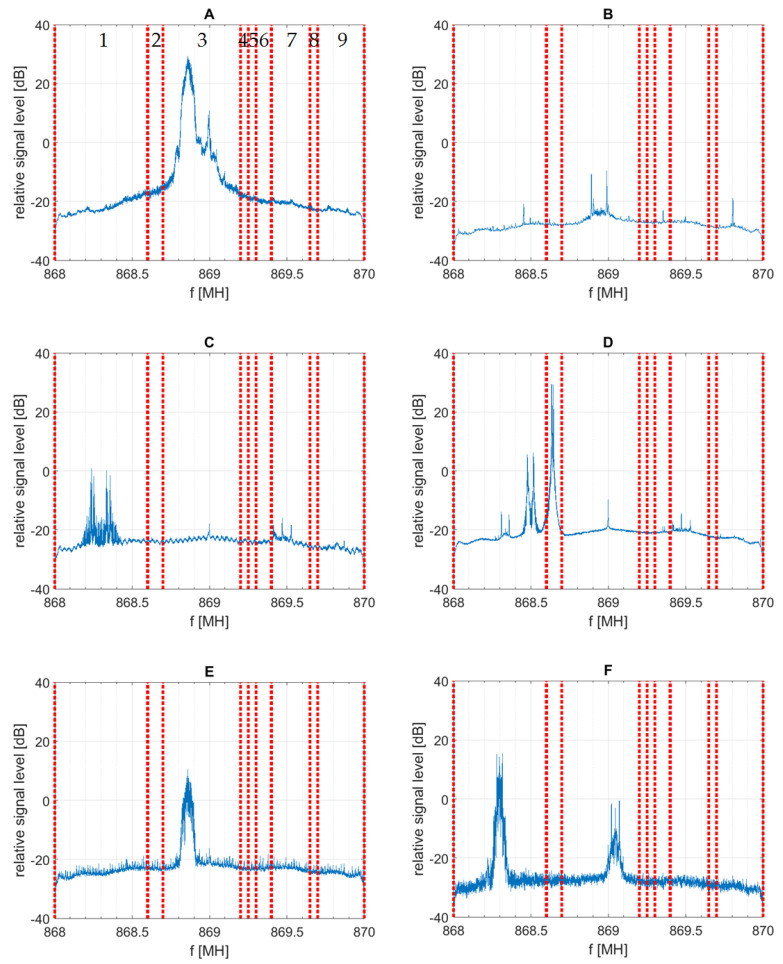
Preliminary test of sub-bands occupancy. (**A**–**F**) refer to the measurement location.

The ISM 868 MHz frequency band is divided into sub-bands, each intended for different applications. Detailed information on this topic is gathered, e.g., in [25]. In this paper, we focus on the range of 868–870 MHz. The band of interest is divided into nine sub-bands. In the first step of the research, the sub-bands were tested for the presence of any signals. The results of those preliminary tests are presented in Figure 3 (dotted lines indicate the division into sub-bands and the numbers added to the spectrum corresponding to location A are sub-band ordinal numbers adopted for the purpose of this paper). Basing on the results obtained, sub-bands listed in Table 1 were selected for further analysis. At this point it should be noticed that the lack of detected transmissions does not rule out the operation of some low duty-cycle system. Accordingly, the empty sub-bands should be considered as intended for further, more careful investigation rather than claimed unoccupied.

**Figure 4:**
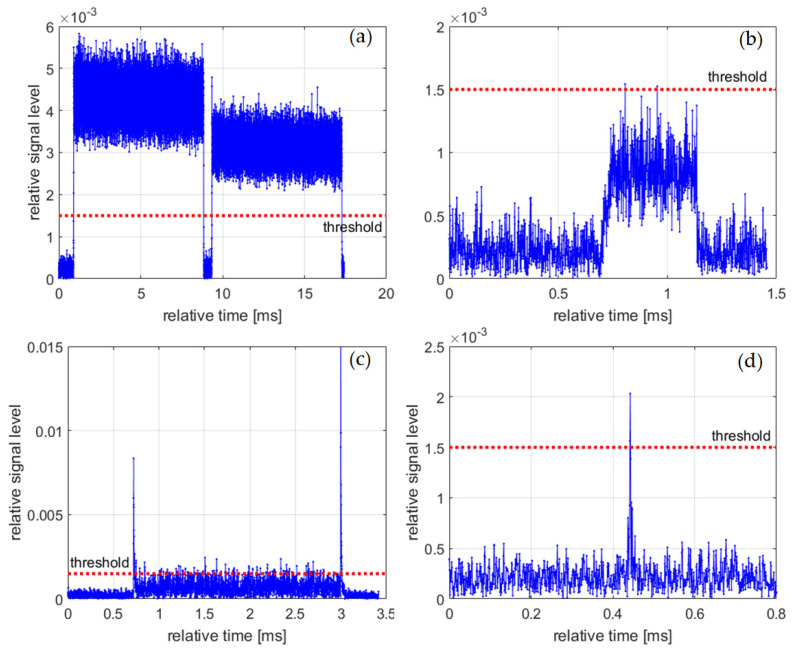
Examples of incorrectly logged bursts and unwanted signals, (**a**) closely spaced bursts; (**b**) a weak burst; (**c**) a burst starting and ending with strong pulses; (**d**) a short pulse.

The analysis of the obtained results revealed that from time to time some misleading information was logged, namely:

1. Two or more closely spaced or even overlapping separate bursts being logged as a single burst;
2. Very weak burst in which only a small number of samples exceed the threshold and therefore neither the start time nor the length are determined correctly;
3. Burst starting and ending with short, strong pulses.
4. Extremely short pulse comprising a few or even only a single sample;

Examples related to the above cases are depicted in Figure 4.

The situation shown in Figure 4a is impossible to distinguish from the correctly performed measurement and will be visible as an untypical, unique burst length in the statistics. 

Burst of the type shown in Figure 4b is likely a signal coming from a distant transmitter. As far as the cases of Figure 4c,d are concerned, it is unlikely that a narrowband system operating in the frequency bands of interest transmits microsecond pulses. The time-domain shapes of the bursts are similar to the ones observed as a result of power leakage from the adjacent bands, i.e., when the signal transmitted in the adjacent band is not properly band-limited. However, the verification of the above hypothesis goes beyond the scope of the paper. 

### 2.5. Data Post-Processing

To remove signals that are clearly not intentional transmissions, the raw measurement data were subjected to post-processing. This consisted of removing all log entries being too short. For a single-carrier system, it can be assumed that the signal having bandwidth of B hertz carries symbols not shorter than 1/B seconds. Since bursts comprising a single symbol are not used, the limit of 10/B was adopted for the post-processing. All shorter log entries were assumed as pulse interferences and removed. Bandwidth B was estimated from the spectra presented in Figure 3.

## 3. Results and Discussion

### 3.1. Channel Occupancy

The channel occupancy is defined as the percentage of the total observation time occupied by the detected bursts. In this paper, the term “channel occupancy” is preferred over “spectrum occupancy”, because the whole sub-band under test is claimed occupied even if only its fragment is used for burst transmission. This can lead to a strong overestimation of the traffic intensity seen by the device using only a part of the sub-band, but on the other hand it allows one to characterize the entire sub-band with a single figure without referring to a particular system and its frequency channel grid.

**Figure 5:**
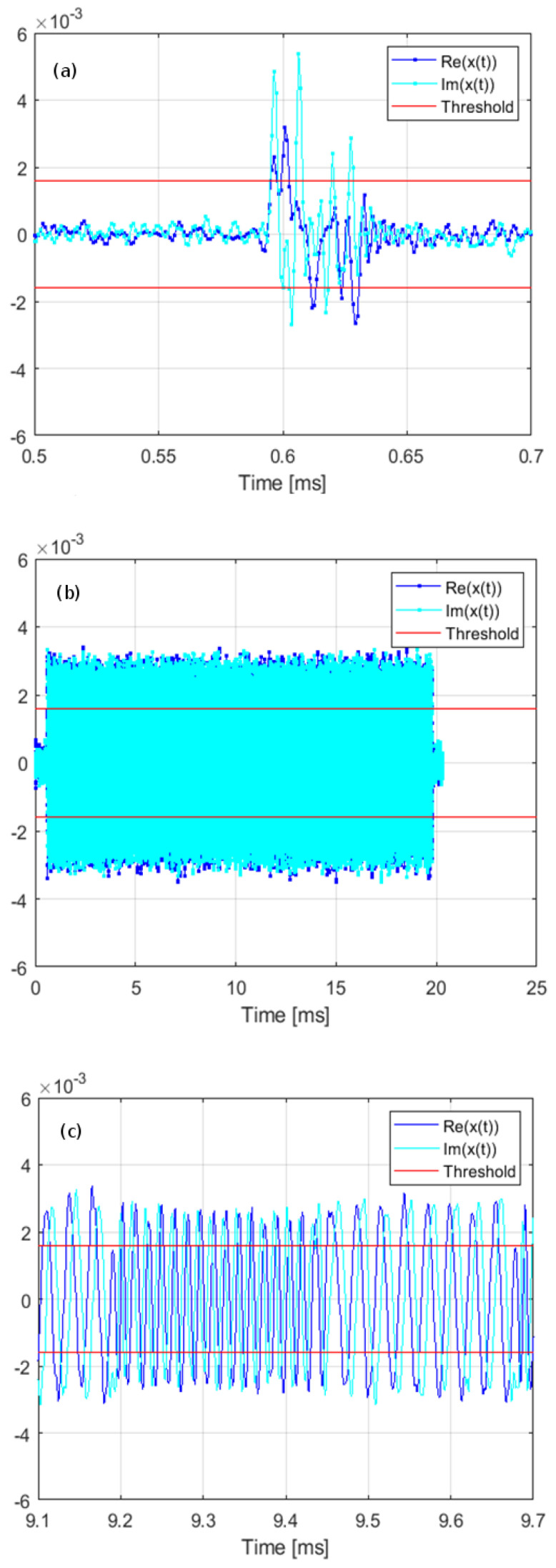
Exemplary bursts received in location C, sub-band 1, (**a**) a burst filtered out during the post-processing; (**b**) a burst considered to be intentionally transmitted; (**c**) a zoomed fragment of the burst shown in part b with frequency modulation visible.

Table 2 presents measured channel occupancy for the raw and post-processed data. As it can be noticed, removing pulse interferences during the post-processing has negligible influence on the results except of the case C1, for which decrease from 0.45% to 0.34% is observed. In-depth analysis showed that for this particular case as many as 240,000 out of 260,000 logged bursts were filtered out due to their length being below the limit of 10/250 kHz = 40 μs. Visual inspection revealed that the typical burst removed by the post-processing looks as presented in Figure 5a, which shows neither fixed amplitude nor internal structure indicating intentional modulation. For contrast, Figure 5b depicts another burst received in the same location and sub-band, having fixed amplitude, a well-defined start and end, and being frequency modulated according to the 2-state FSK scheme, clearly visible after zooming in on Figure 5c.

Table 2 shows that the measured channel occupancy is surprisingly low, compared, e.g., with [25]. This may be due to their different locations. However, it should be stressed that the comparison of the results obtained using different measurement methodologies may be misleading. On the one hand, the measurement set-up used provides a high time resolution equal to the sampling interval, which is a maximum of 5 μs. In consequence, a burst length is measured accurately, in contrast to experiments in which time bins were analyzed and a whole time bin was considered occupied regardless of an actual burst length if only the burst carried enough power to be detected. On the other hand, analyzing the sub-band as a whole is equivalent to poor frequency resolution which, as it has been already mentioned above, may lead to an overestimation of the result.

### 3.2. Transmission Identification

**Figure 6:**
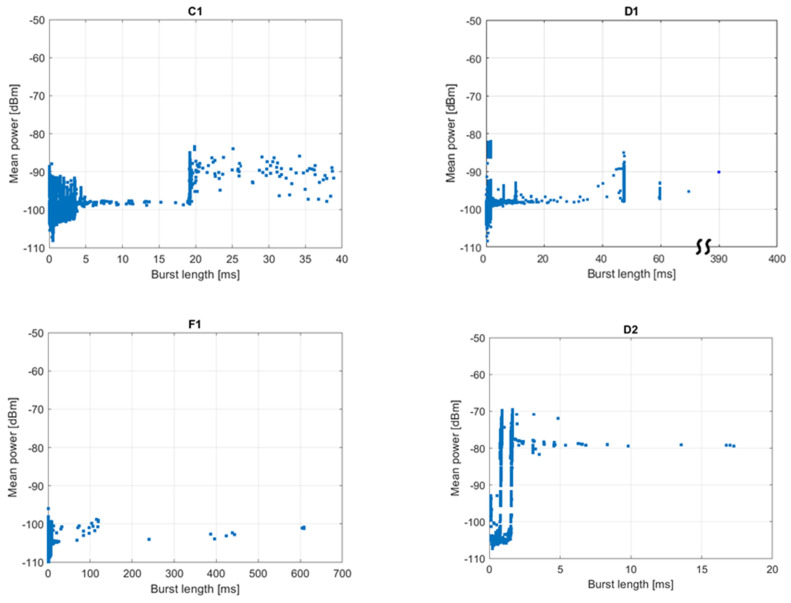

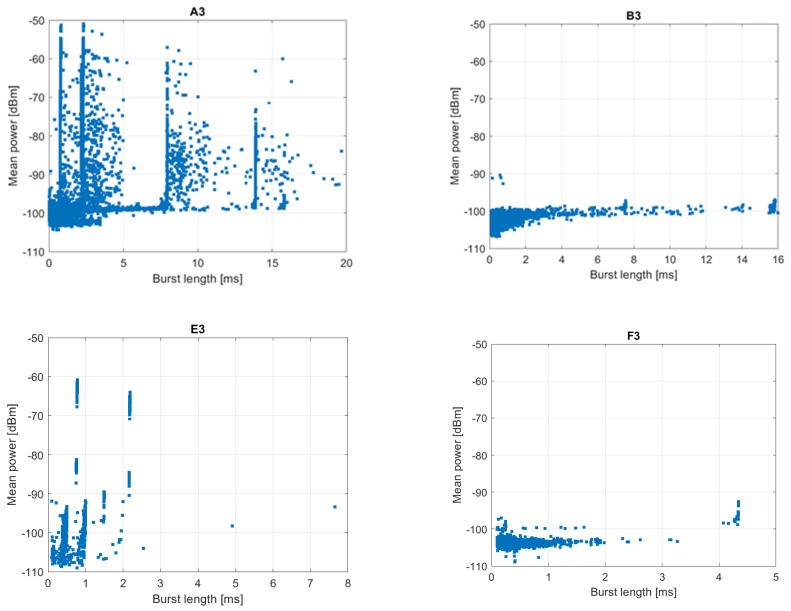
Post-processed measurement results on the power-length plane. The labels C1 to F3 above the plots denote the measurement location and sub-band.

Figure 6 shows the post-processed measurement results on the power-length plane. In such a plot, a non-mobile system transmitting bursts of fixed length should leave a trace in the form of a single dot. However, one can notice that vertical lines appear instead which means that bursts of the same length were received at different power levels. The differences may be as high as 50 dB (see plot for location A, sub-band 3). This phenomenon can be explained in several ways:

- A number of transmitters located at different distances from the measurement set-up emit bursts of the same length;
- Transmitters are mobile or portable;
- Propagation channel attenuation fluctuates because the apartments under test are used as usual, i.e., people are moving, doors are opened and closed, etc.

Collected data do not give an answer which ones of the above factors had the decisive influence on the power fluctuations. 

Based on Figure 6, an attempt to identify intentional transmissions was made by reading the positions of the vertical lines. The results are presented in Table 3. For most of the tested sub-bands, several well-defined burst lengths were found; however, their number should not be considered a number of individual systems operating in a given location under testing. For example, more detailed analysis reveals that in sub-band 3, in both locations A and E, a 0.8 ms long burst follows a 2.2 ms long one with a time separation of 17.3 ms. This leads to the conclusion that they are transmitted by a single device according to some communication protocol. Additionally, some bursts detected in location D in sub-band 1 were identified as being inter-channel interferences from sub-band 2, so they were detected and logged twice. This issue is described in more detail in case studies presented in Section 3.3.

### 3.3. Case Study—Location D

Many relationships between individual bursts can be determined on the basis of the analysis of burst lengths as a function of time of observation. Since it is not possible to present such an analysis for all sub-bands and locations in a single paper, we will limit ourselves to the cases of D1 and D2, for which the most clear transmission patterns were found.

**Figure 7:**
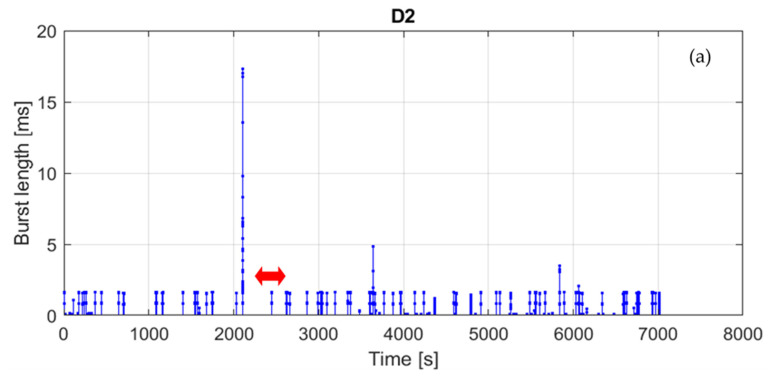

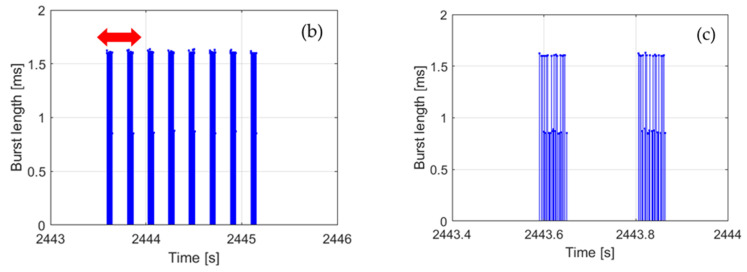
Measurement results for location D, sub-band 2: (**a**) whole measurement duration, (**b**) zoomed bursts indicated by the arrow in part a; (**c**) zoomed bursts indicated by the arrow in part b.

Figure 7 presents the post-processed measurement results for location D and sub-band 2, expressed as the burst length against burst start time in relation to the beginning of the measurement. It can be observed that most of the traffic in the sub-band consists of series of eight groups of alternately occurring 0.8 and 1.6 ms long pulses. The duration of a single group equals approx. 1.5 s. This observation is also confirmed in Figure 6, in which a certain number of points are grouped into two vertical lines at 0.8 and 1.6 ms. Since the groups are distributed over the whole observation time, the corresponding duty cycle cannot be greater than the channel occupancy, i.e., 0.26%. Other bursts show no obvious regularity, neither in Figure 6 nor in Figure 7.

**Figure 8:**
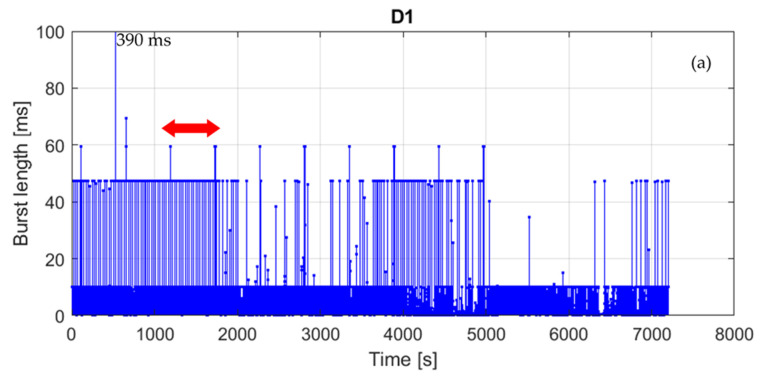

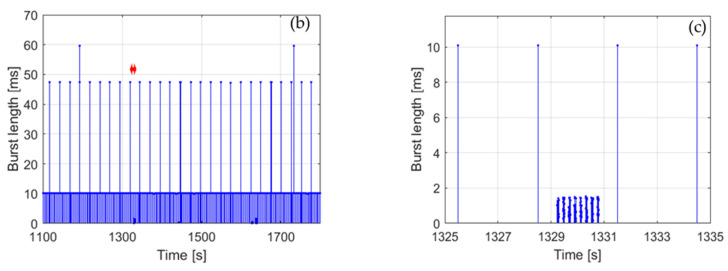
Measurement results for location D, sub-band 1: (**a**) whole measurement duration, (**b**) zoomed bursts indicated by the arrow in part a; (**c**) zoomed bursts indicated by the arrow in part b.

In sub-band 1 (Figure 8) at least four types of transmission can be identified in addition to a single very long (390 ms) burst of an unknown origin. The first transmission consists of groups of 60 ms-long bursts emitted with the period of approx. 540 s, which corresponds to the duty cycle of 0.01%. The second one consists of 47 ms long bursts emitted with the period of 25.25 s, although the periodicity is from time to time disturbed by the occurrence of additional bursts. In this case, the duty cycle equals 0.2%. The third transmission consists of 10 ms long bursts emitted every 3 s (duty cycle 0.3%). Finally, the last type of transmission, shown in Figure 8c, is the same as detected in sub-band 2, although it is received at much lower power levels. Basing on the above, one can presume that the last transmission is just an interchannel interference from sub-band 2. This leads to the conclusion that devices operating in the band of interest are not always well-designed in terms of frequency selectivity and may influence on other devices operating in adjacent channels or at least artificially inflate adjacent channels’ occupancy, which may result in increased channel access problems.

### 3.4. Medium Accesibility

Systems operating in the frequency bands of interest are not time-synchronized with each other. Moreover, synchronization within a single system is also usually not provided. The transmitters emit bursts in random time instances and the probability of bursts collision is kept low by low duty cycles of particular systems. Such kind of medium access control works well if the number of transmitters using the same channel is limited. The more transmitters operate on the same frequency, the more collisions occur, and at some point the number of collisions becomes unacceptable. On the basis of the obtained channel occupancy values only, one can deduce that in all cases collisions are unlikely and more systems may be run in the tested frequency bands. However, since the logged data provide also information about bursts distribution over time, it was possible to analyze idle time accessible for possible additional transmitters in more details. 

**Figure 9:**
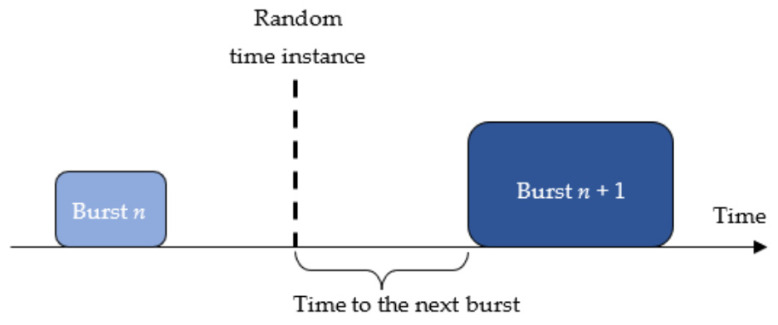
The definition of the time to the next burst.

Figure 9 defines a parameter referred to as “time to the next burst” as a continuous block of idle time to be used by a hypothetical additional transmitter activated in a random time instance. Table 4 shows median and the 10th percentile of the time to the next burst determined on the basis of the measurement results using Monte Carlo simulations. The median indicates the maximum length of a burst that can be transmitted via tested channel with a success rate of 50%, whereas the 10th percentile corresponds to a success rate of 90%.

The simulation results range from tens of milliseconds to seconds (10th percentile) or tens of seconds (median), depending on the location, sub-band, and whether the post-processing is performed or not. Turning the post-processing on is equivalent to making an assumptions that a short pulse is an interference carrying no useful data and it does not disturb the narrowband signal to the extent that makes the reception impossible. The latter is of course only true for the reasonable ratio of the pulse and signal power.

The values gathered in Table 4 should be compared with the expected duration of a single act of communication in the frequency range under test. Although according to Table 3 the length of the burst is expressed in milliseconds or tens of milliseconds, it was shown in the previous section that the bursts may be grouped together to form a message. In the case study corresponding to sub-band 2 and location D, the message lasting for 1.5 s in total was identified. SigFox messages last for up to 2 s, and LoRa messages up to about 0.9 s [26]. Considering the above, it can be concluded that switching on another transmitter may be more problematic than it would appear from the channel occupancy values. For example, in the case of D1 about half of the attempts of transmitting a 1.5 s long message will fail, although the channel occupancy is only 0.54%. This is due to the presence of signals emitted every 3 s, although they use only 0.3% of the total channel time.

## 4. Conclusions

In this study, a self-constructed SRD-based measurement set-up was used to monitor part of the 868 MHz ISM frequency band for the presence of transmissions. The measurements were carried out in six apartments located in residential areas of Warsaw, Poland. The performed monitoring covered frequencies from 868 MHz to 870 MHz. In general, activity was detected below 869.2 MHz, whereas the sub-bands above 869.2 MHz appeared unoccupied in all six measurement locations with the caveat that certain low duty cycle transmissions may have been missed.

Detailed analysis of the sub-bands below 869.2 MHz showed that the channel occupancy is usually below 1%, with about 2% in one case. In all occupied sub-bands and all locations, both intentional transmissions and unintentional interferences were detected. In particular, at least one signal having properties of power leakage from the adjacent sub-band was identified. This leads to the conclusion that devices operating in the frequency range of interest are not always well designed in terms of frequency selectivity, which may increase problems with medium access by artificially inflating the channel’s occupancy.

Monte Carlo simulations showed that under the conditions observed during the measurement campaign, continuous idle time blocks available for new transmitters may be insufficient for collision-free operation. This is due to the presence of the signals which are transmitted at short time intervals, but are short enough to not to violate the low duty cycle rule. It follows that the channel availability for potential new systems should not be estimated solely on the basis of the channel occupancy value.

## Figures and Tables

**Table 1:** Sub-bands selected for further analysis.

Location	Sub-Band No.	Sub-Band Boundaries [MHz]
C	1	868.00–868.60
D	1	
F	1	
D	2	868.60–868.70
A	3	868.70–869.20
B	3	
E	3	
F	3	

**Table 2:** Channel occupancy for raw and post-processed data.

Location/Sub-Band	Occupancy [%] (Raw)	Occupancy [%] (Post-Processed)
C1	0.45	0.34
D1	0.54	0.54
F1	0.29	0.29
D2	0.26	0.26
A3	2.06	2.06
B3	0.072	0.072
E3	0.015	0.015
F3	0.012	0.012

**Table 3:** Identified burst lengths.

Location/Sub-Band	Burst Length [ms]
C1	0.5, 1, 1.5, 2, 2.5, 3, 3.5, 4.3, 19
D1	0.8, 1.6, 10, 47, 60
F1 ^1^	
D2	0.8, 1.6
A3	0.8, 2.2, 2.3, 8, 14
B3 ^1^	
E3	0.5, 0.8, 1, 1.5, 2.2
F3 ^2^	4.3

^1^ No bursts having properties of intentional transmission detected. ^2^ Doubtful observation due to low power.

**Table 4:** Time to the next burst in [s].

Location/ Sub-Band	Median		10th Percentile	
	Raw	Post- Processed	Raw	Post- Processed
C1	0.09	1.8	0.003	0.11
D1	1.4	1.5	0.23	0.25
F1	2.0	2.3	0.35	0.43
D2	9.80	44.4	1.4	5.4
A3	0.11	0.12	0.012	0.013
B3	2.1	6.7	0.078	0.72
E3	13.9	16.1	1.87	2.67
F3	6.8	21.5	0.55	2.1

## Data Availability

The logs being the base of the presented analysis might be available from the authors upon reasonable request.

## References

[1] Chen X., Ng D.W.K., Yu W., Larsson E.G., Al-Dhahir N., Schober R. (2021). Massive Access for 5G and Beyond. IEEE J. Sel. Areas Commun..

[2] Jiang X., Zhang H., Yi E.A.B., Raghunathan N., Mousoulis C., Chaterji S., Peroulis D. (2020). Hybrid Low-Power Wide-Area Mesh Network for IoT Applications. IEEE Internet Things J..

[3] Solic P., Colella R., Catarinucci L., Perkovic T., Patrono L. (2019). Proof of Presence: Novel Vehicle Detection System. IEEE Wirel. Commun..

[4] Catherwood P.A., Steele D., Little M., McComb S., McLaughlin J. (2018). A Community-Based IoT Personalized Wireless Healthcare Solution Trial. IEEE J. Transl. Eng. Health Med..

[5] Tseng K.-H., Chung M.-Y., Chen L.-H., Chang P.-Y. (2021). Green Smart Campus Monitoring and Detection Using LoRa. Sensors.

[6] Ojo M.O., Adami D., Giordano S. (2021). Experimental Evaluation of a LoRa Wildlife Monitoring Network in a Forest Vegetation Area. Future Internet.

[7] Saelens M., Hoebeke J., Shahid A., De Poorter E. (2019). Impact of EU duty cycle and transmission power limitations for sub-GHz LPWAN SRDs: An overview and future challenges. J. Wirel. Commun. Netw..

[8] Sanders F.H. Broadband spectrum surveys in Denver, CO, San Diego, CA, and Los Angeles, CA: Methodology, analysis, and comparative results. Proceedings of the 1998 IEEE EMC Symposium. International Symposium on Electromagnetic Compatibility.

[9] Islam M.H., Koh C.L., Oh S.W., Qing X., Lai Y.Y., Wang C., Liang Y.-C., Toh B.E., Chin F., Tan G.L. Spectrum Survey in Singapore: Occupancy Measurements and Analyses. Proceedings of the 3rd International Conference on Cognitive Radio Oriented Wireless Networks and Communications (CrownCom 2008).

[10] (2010). General Survey of Radio Frequency Bands—30 MHz to 3 GHz. Shared Spectrum Company Report. https://www.sharedspectrum.com/wp-content/uploads/2021/01/2010_0923-General-Band-Survey-30MHz-to-3GHz.pdf.

[11] Ramirez D.A.A., Cardenas-Juarez M., Pineda-Rico U., Arce A., Stevens-Navarro E. Spectrum Occupancy Measurements in the Sub-6 GHz Band for Smart Spectrum Applications. Proceedings of the 2018 IEEE 10th Latin-American Conference on Communications (LATINCOM).

[12] Chen Y., Oh H.S. (2016). A Survey of Measurement-Based Spectrum Occupancy Modeling for Cognitive Radios. IEEE Commun. Surv. Tutor..

[13] Li H., Liu L., Li Y., Yuan Z., Zhang K. (2019). Measurement and Characterization of Electromagnetic Noise in Edge Computing Networks for the Industrial Internet of Things. Sensors.

[14] Jamoos A., Abdou A. Spectrum Measurements and Analysis for Cognitive Radio Applications in Palestine. Proceedings of the 6th International Conference on Electrical and Electronics Engineering (ICEEE).

[15] Chantaveerod A., Woradit K., Pochaiya C. (2021). Spectrum Occupancy Model Based on Empirical Data for FM Radio Broadcasting in Suburban Environments. Sensors.

[16] Aygül M.A., Nazzal M., Sağlam M.İ., da Costa D.B., Ateş H.F., Arslan H. (2021). Efficient Spectrum Occupancy Prediction Exploiting Multidimensional Correlations through Composite 2D-LSTM Models. Sensors.

[17] Cardenas-Juarez M., Diaz-Ibarra M.A., Pineda-Rico U., Arce A., Stevens-Navarro E. On spectrum occupancy measurements at 2.4 GHz ISM band for cognitive radio applications. Proceedings of the International Conference on Electronics, Communications and Computers (CONIELECOMP).

[18] Rademacher M., Jonas K., Kretschmer M. Quantifying the spectrum occupancy in an outdoor 5 GHz WiFi network with directional antennas. Proceedings of the 2018 IEEE Wireless Communications and Networking Conference (WCNC).

[19] Cheema A.A., Salous S. (2019). Spectrum Occupancy Measurements and Analysis in 2.4 GHz WLAN. Electronics.

[20] Mostahinic N., Refai H. Spectrum Occupancy for 802.11a/n/ac Homogeneous and Heterogeneous Networks. Proceedings of the 15th International Wireless Communications & Mobile Computing Conference (IWCMC).

[21] Kumar K.S., Lee Y.H., Meng Y.S. Spectrum Survey in 2.4 GHz ISM Band for a MRT Station Located in Singapore. Proceedings of the IEEE USNC-CNC-URSI North American Radio Science Meeting (Joint with AP-S Symposium).

[22] Mucchi L., Vuohtoniemi R., Virk H., Conti A., Hämäläinen M., Iinatti J., Win M.Z. (2020). Spectrum Occupancy and Interference Model Based on Network Experimentation in Hospital. IEEE Trans. Wirel. Commun..

[23] Barrachina-Muñoz S., Bellalta B., Knightly E.E. (2021). Wi-Fi Channel Bonding: An All-Channel System and Experimental Study from Urban Hotspots to a Sold-Out Stadium. IEEE/ACM Trans. Netw..

[24] Lauridsen M., Frederiksen F., Rodriguez I. Feasibility of deploying wireless Internet of Things in the unlicensed European 865–868 MHz band. Proceedings of the IEEE Wireless Communications and Networking Conference (WCNC).

[25] Lauridsen M., Vejlgaard B., Kovacs I.Z., Nguyen H., Mogensen P. Interference Measurements in the European 868 MHz ISM Band with Focus on LoRa and SigFox. Proceedings of the IEEE Wireless Communications and Networking Conference (WCNC).

[26] Vejlgaard B., Lauridsen M., Nguyen H., Kovacs I.Z., Mogensen P., Sørensen M. Interference Impact on Coverage and Capacity for Low Power Wide Area IoT Networks. Proceedings of the IEEE Wireless Communications and Networking Conference (WCNC).

[27] Lieske H., Beer F., Kilian G., Robert J., Heuberger A. Characterisation of Channel Usage in ISM/SRD Bands. Proceedings of the 34th European Telemetry and Test Conference (ETC).

[28] National Instruments Support, USRP-2932. https://www.ni.com/pl-pl/support/model.usrp-2932.html.

[29] Frolik J., Golmohamadi M. (2017). On Random and Multidimensional Channel Effects in Cluttered Environments. IEEE Antennas Wirel. Propag. Lett..

[30] GNU Radio Home Page. https://www.gnuradio.org.

